# Simulation of population dynamics of *Bulinus globosus*: Effects of environmental temperature on production of *Schistosoma haematobium* cercariae

**DOI:** 10.1371/journal.pntd.0006651

**Published:** 2018-08-02

**Authors:** Chester Kalinda, Moses J. Chimbari, William E. Grant, Hsiao-Hsuan Wang, Julius N. Odhiambo, Samson Mukaratirwa

**Affiliations:** 1 School of Nursing and Public Health, College of Health Sciences, Howard College Campus, University of KwaZulu-Natal, Durban, South Africa; 2 Ecological Systems Laboratory, Department of Wildlife and Fisheries Sciences, College of Agriculture and Life Sciences, Texas A&M University, College Station, Texas, United States of America; 3 School of Life Sciences, College of Agriculture, Engineering and Science, Westville Campus, University of KwaZulu-Natal, Durban, South Africa; Institute of Tropical Medicine (NEKKEN), Nagasaki University, JAPAN

## Abstract

**Background:**

Temperature is an important factor that influences the biology and ecology of intermediate host (IH) snails and the schistosome parasites they transmit. Although temperature shifts due to climate change has been predicted to affect the life history traits of IH snails and parasite production, the mechanisms of how this may affect parasite abundance and disease risks are still not clear.

**Materials and methods:**

Using data from laboratory and field experiments, we developed a deterministic compartmental simulation model based on difference equations using a weekly time step that represented the life cycle of *Bulinus globosus*. We simulated snail population dynamics and the associated production of cercariae assuming current environmental temperatures as well as projected temperature increases of 1 °C and 2 °C.

**Results:**

The model generated snail fecundity and survival rates similar to those observed in the laboratory and also produced reasonable snail population dynamics under seasonally varying temperatures representative of generally favorable environmental conditions. Simulated relative abundances of both snails and cercariae decreased with increasing environmental temperatures, with maximum snail abundances decreased by 14% and 27%, and maximum cercariae productions decreased by 8% and 17%, when temperatures were increased by 1 °C and 2 °C, respectively.

**Conclusion:**

The results indicate that future rise in temperature due to climate change may alter the abundance of *B*. *globosus* and impact on the prevalence of schistosomiasis. Furthermore, increased temperatures may not linearly influence the abundance of *S*. *haematobium*. These results may have important implications for schistosomiasis control programmes in view of temperature driven changes in the life history traits of *B*. *globosus* and *S*. *haematobium*. Our study recommends that the use of deterministic models incorporating the effects of temperature on the life history traits of IH snails would be vital in understanding the potential impact of climate change on schistosomiasis incidences and prevalence.

## Author summary

The implementation of schistosomiasis control/elimination strategies depend on accurate estimations and predictions of the changes in the snail and parasite population. This is essential especially in the allocation of limited resources. The simulation model and the results presented here provide useful information on *Bulinus globosus*, the main intermediate host snail of *Schistosoma haematobium* in southern Africa. The predicted changes in the abundance of *B*. *globosus* and *S*. *haematobium* due to changes in temperature may be vital in the fight against schistosomiasis. The model predicts the current and future abundance of intermediate hosts snails by considering the future predicted temperature increases. These results may be useful in evaluating the snail-trematode interactions within the natural systems in which changes in the environmental conditions such a temperature may affect the population size of *Bulinus globosus* and disease incidences. These effects of temperature on *B*. *globosus* may play an important role in the implementation of snail control programmes.

## Introduction

Growing evidence suggests that climate change may alter the distribution and prevalence of infectious diseases and this may have detrimental effects on human health [1, 2]. Change in climate has been predicted to lead to a rise in temperature, thus creating suitable habitats for various freshwater intermediate host (IH) snails [3, 4]. In contrast, Stensgaard et al. [5] suggested a possible overall contraction in suitable areas for IH snails, especially those that transmit *S*. *mansoni* in Africa. Rise in temperature has also been suggested to increase intramolluscan parasite development rates [6–8] and output [9, 10]. Nevertheless, high temperatures have been associated with increased snail mortality [11–13] thus increasing the challenges associated with predicting its net effect on disease risks [10].

The development of *Schistosoma haematobium* within its IH snail, *Bulinus globosus*, has been described by Appleton and Madsen [14] while its biology and ecology has been extensively studied [8, 15, 16]. While rise in temperature has been predicted to alter the biology and ecology of IH snails [4, 5, 7, 8, 17], the prospects of range change, reductions or expansions for *B*. *globosus* and risks posed by *S*. *haematobium* infections presently remain unclear. This is because the production and distribution of *S*. *haematobium* may be affected by the thermal limits (low and high temperatures) that determine the physiological processes of *B*. *globosus* [2, 18]. For instance, at lower temperatures, parasite development is reduced and snail growth is inhibited [19, 20] while snail survival is high [8]. In contrast, at high temperature levels, snail mortality is high [11, 21] and parasite development reduced [22].

The complexity of the life cycle of *Schistosoma* and its size may make it adapt faster to temperature shifts than its IH snails [23] suggesting a possible shift in the risks of *Schistosoma* transmission with rise in temperature. Such phenological differences between parasites and their hosts may compound the challenges associated with predicting the net impact of climate change on *S*. *haematobium* distribution and output [23, 24]. However, simulation models based on laboratory and field experiments provide flexible and useful methods for incorporating various complex interactions between IH snails and environmental factors such as temperature, to evaluate the overall impact on host population dynamics and disease risks [25].

The exact mechanisms of how climate change is likely to alter the prevalence and risks of *Schistosoma* infection has become an urgent public health issue as evidenced by the number of studies that have explored this [4, 6–8, 26–29]. A study by Mccreesh and Booth [30] suggested that temperature shifts may lead to altered infection risks especially during seasons of low and high temperatures. However, their model assumed that all infected snails move through the pre-patent period to the patent period to produce cercariae which may not be the case [8, 21]. In order to understand how increasing water temperatures may affect the transmission of schistosomiasis, we examined the effects of environmental temperature on *B*. *globosus* population dynamics and the production of *S*. *haematobium* cercariae using a simulation model adapted from that of Marín et al. [25]. We hypothesized that rise in temperature which has altered the geographical spread of IH snails [4, 17] and created more micro-habitats [3, 27] would lead to increased parasite output and snail population size.

## Materials and methods

### Model description

**Fig 1:**
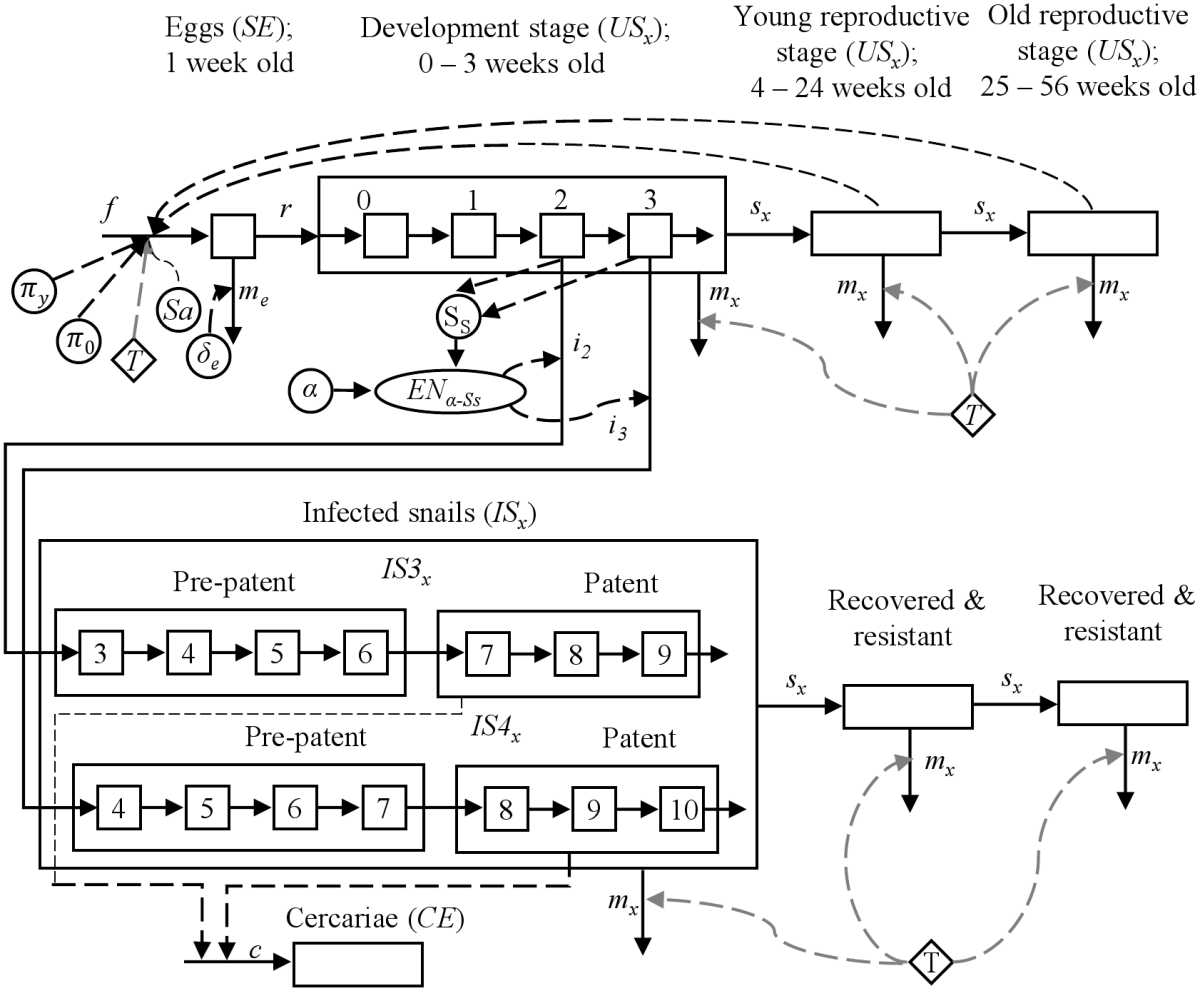
Conceptual model representing the life cycle and infection of snails and parasite development within snails. *SE* represents snail eggs; *US*_*x*_ represents uninfected snails population at *x* weeks of age; *IS*_*x*_ represents infected snails population at *x* weeks of age; *CE* represents the cercariae; *f* represents fecundity; π_y_ represents the number of eggs per mass produced by young (4 ≤ x ≤ 24 weeks) snails; π_o_ represents the number of eggs per mass produced by old (25 ≤ x ≤ 56 weeks) snails; *T* represents temperature; *Sa* represents snail age; *δ*_*e*_ represents egg mortality rate; *m*_*e*_ represents egg mortality; *r* represents recruitment; *S*_*s*_ represents susceptible snails; *α* represents parasite eggs; *EN*_*α-Ss*_ represents infection rate; *i*_*2*_ and *i*_*3*_ are the number of snails becoming infected from the state variables representing the number of snails at 2 and 3 weeks of age; *m*_*x*_ represents mortality; *s*_*x*_ represents age-specific survival; bold dash line represents temperature effects.

The model represents snail (*B*. *globosus)* population dynamics, snail infection, and parasite development in the snail (Fig 1). The parasite goes through a series of intra-snail stages before being released as a free-living stage (cercariae). The main processes affecting parasites are recruitment of susceptible snails, snail infection, snail mortality, and the shedding of cercariae. Recruitment depends on temperature and number of reproductively mature snails (ages 4–56 weeks). Infection depends on number of susceptible snails (ages 2–3 weeks) and availability of miracidia. Mortality depends on temperature and stage of snail development. Shedding of cercariae depends on number of infected snails in patent stages (5–7 weeks post infection).

The model is a deterministic compartmental model based on difference equations using a weekly time step, which we programmed using STELLA software (High Performance Systems Inc.). In the description that follows, state variables are denoted by double upper-case letters, driving variables are denoted by a single upper-case letter, material transfers are denoted by a single lower-case letter, and parameters are denoted by a single lower case Greek letter. Auxiliary variables are used to define material transfers that need information elsewhere in the model, and are denoted by an upper and a lower-case letter. Some of the equations and parameter values have been adopted from Marín et al. [25], except for the water temperature driving variable values, the representation of earlier ages at which egg masses are first produced, and the temperature effects of the number of egg masses laid and on snail mortality, which are based on Kalinda et al. [8]. The values of parameters and associated information sources are presented in Table 1. All parameter values taken from Marín et al. [25], except for those referred to in the text concerning the earlier age at which egg masses are produced and the water temperature effects on the number of egg masses produced and snail mortality.

**Table 1:** Summary of the quantitative information used in the model representing parasite eggs in the environment, snail population dynamics, and parasite development within the snail.

Parameter	Value	Source
α	12 600	Dronen [31]
δ_e_	0.200	Dewitt [32]
π_y_	8	Fernandez and Esch [33]
π_o_	41	Laboratory observations
ω	2100	Dronen [31]

Values of rates are expressed per week. α, number of eggs produced per adult parasite. δ_e_, proportion of snail eggs that die. π_y_, number of eggs per mass produced by young (4 ≤ x ≤ 24 weeks) snails. π_o_, number of eggs per mass produced by old (25 ≤ x ≤ 56 weeks) snails. ω, number of cercariae released into the environment per week.

The snail population was divided into uninfected (*US*_*x*_) and infected portions (*IS*_*x*_), the latter shedding cercariae into the environment (*c*). The snail population was divided into 1-week age classes to represent age-specific differences in both susceptibility to infection and rates of parasite development in infected snails. Material transfers into and out of the state variables represent snail fecundity (*f*), affected by snail age (*Sa*) and temperature (*T*); survival of snail eggs (*SE*), represented as recruitment (*r*); egg mortality (*m*_*e*_); age-specific survival (*s*_*x*_) and mortality (*m*_*x*_) of snails, with the latter being affected by temperature. The number of snails becoming infected was represented by two additional material transfers coming from the state variables representing the number of snails at 2 and 3 weeks of age (*i*_*2*_ and *i*_*3*_, respectively), which constituted the susceptible portion of the snail population (Fig 1).

Material transfers, *i*_*2*_ and *i*_*3*_ also correspond to the input of snails to two different series of state variables representing the subsequent development of infected snails (*IS3*_*x*_ and *IS4*_*x*_). Infection rate was a function of the number of encounters (*EN*_*α-Ss*_) between susceptible snails and parasite eggs, which depends on the abundance of both parasite eggs (α) and susceptible snails (*Ss*). By keeping those snails infected at 2 weeks of age distinct from those infected at 3 weeks of age, it was possible to distinguish snails having pre-patent infections (infected snails not shedding cercariae) from snails having patent infections (infected snails shedding cercariae into the environment) [34]. In order to estimate of the input of cercariae to the environment (*c*), the duration of pre-patent and patent infections were set at 3 weeks and 4 weeks, respectively [31, 35]. Equations describing the state variables of snail sub-model are:

$$SE_{t+1}=SE_{t}+f-m_{e}-r$$ (1)

$$US_{x,t+1}=US_{x,t}+s_{x-1}-m_{x}-s_{x}\;\text{for}\;\text{all}\;\text{x}\;\text{except}\;\text{x}=2\;\text{and}\;\text{x}=3$$ (2)

$$US_{x,t+1}=US_{x,t}+s_{x-1}-m_{x}-s_{x}-i_{x}\;\text{for}\;\text{x}=2\;\text{and}\;\text{x}=3$$ (3)

$$IS_{x,t+1}=IS_{x,t}+i_{x-1}-m_{x}-s_{x}\;\text{for}\;\text{x}=3\;\text{and}\;\text{x}=4$$ (4)

$$IS_{x,t+1}=IS_{x,t}+s_{x-1}-m_{x}-s_{x}\;\text{for}\;\text{all}\;x>4$$ (5)

The driving variable of this model was weekly temperature in °C. The material transfer describing the number of eggs produced by sexually mature snails (*f*) was represented as:

$$f=\left(\sum_{from\;x=4\;to\;x=24}US_{x,t}+\sum_{from\;x=4\;to\;x=24}IS_{x,t}\right)*\varsigma *\pi_{y}+\left(\sum_{from\;x=25\;to\;x=56}US_{x,t}+\sum_{from\;x=25\;to\;x=56}IS_{x,t}\right)*\varsigma *\pi_{o}$$ (6)

where *ς* is the number of egg masses produced per snail, *π*_*y*_ and *π*_*o*_ are the numbers of eggs per mass produced by young (4 weeks ≤ x ≤ 24 weeks) and old snails (25 weeks ≤ x ≤ 56 weeks), respectively. The age at which egg masses are first produced was based on data in Kalinda et al. [8]. The temperature effect on the number of egg masses produced per snail per week was represented as:

ς=−0.0012*T^3+0.0642*T^2−0.7827*T+1.1353 (7)

where *T* is water temperature in degrees C. The number of snail eggs that die (*m*_*e*_) and survive to develop into snails (*r*) was represented as:

$$m_{e}=\delta_{e}*SE_{t}$$ (8)

$$r=SE_{t}-m_{e}$$ (9)

where *δ*_*e*_ is the proportion of snail eggs that die. Material transfers representing age-specific mortality (*m*_*x*_) and survival (*s*_*x*_) of uninfected snails (*US*_*x*_) were represented as:

$$m_{x}=\delta_{x}*US_{x,t}\;\text{for}\;\text{all}\;\text{x}\;\text{except}\;\text{x}=2\;\text{and}\;\text{x}=3$$ (10)

$$s_{x}=US_{x,t}-m_{x}\;\text{for}\;\text{all}\;\text{x}\;\text{except}\;\text{x}=2\;\text{and}\;\text{x}=3$$ (11)

$$m_{x}=\left(US_{x,t}-i_{x}\right)*\delta_{x}\;\text{for}\;\text{x}=2\;\text{and}\;\text{x}=3$$ (12)

$$s_{x}=US_{x,t}-i_{x}-m_{x}\;\text{for}\;\text{x}=2\;\text{and}\;\text{x}=3$$ (13)

where *δ*_*x*_ is proportion of uninfected snails that die during age x and *i*_*x*_ is the number of snails of age x becoming infected.

Material transfers representing age-specific mortality (*m*_*x*_) and survival (*s*_*x*_) of infected snails (*IS*_*x*_) were represented as:

$$m_{x}=\delta_{x}*IS_{x,t}\;\text{for}\;\text{all}\;\text{x}\;\text{except}\;\text{x}=2\;\text{and}\;\text{x}=3$$ (14)

$$s_{x}=IS_{x,t}-m_{x}\;\text{for}\;\text{all}\;\text{x}\;\text{except}\;\text{x}=2\;\text{and}\;\text{x}=3$$ (15)

$$m_{x}=\;\left(IS_{x,t}+i_{x}\right)\;*\delta_{x}\;\text{for}\;\text{x}=2\;\text{and}\;\text{x}=3$$ (16)

$$s_{x}=IS_{x,t}+i_{x}-m_{x}\;\text{for}\;\text{x}=2\;\text{and}\;\text{x}=3$$ (17)

where *δ*_*x*_ is the proportion of uninfected snails that die during age x. In this model it was assumed that parasites have no effect on snail survival [19] and that temperature had the same effect on survival of snails of all ages past the egg stage, therefore the same value of *δ*_*x*_ is used for both uninfected and infected snails of all ages past the egg stage. The effect of water temperature on *δ* was represented as:

δ=0.0001*e^(0.246*T) (18)

where *T* is water temperature in degrees C.

Material transfer representing input of cercariae to the environment (*c*) was represented as:

$$c=\left(\sum_{from\;x=7\;to\;x=9}IS3_{x,t}+\sum_{from\;x=8\;to\;x=10}IS4_{x,t}\right)*\omega$$ (19)

where ω is the number of cercariae released into the environment per infected snail.

### Model evaluation

To evaluate model performance, we first verified that the model code was capable of generating snail fecundity (egg masses produced per snail per week) and survival (proportion surviving) rates similar to those observed over a range of water temperatures in the laboratory [10]. We then assessed the ability of the model to simulate snail population dynamics under seasonally varying environmental temperatures representative of the general conditions identified as favorable by Manyangadze et al. [3]. Note that we refer to model verification, following Rykiel [36], as a demonstration that the model formalism is correct, that there are no errors in the model code or logic. We refer to model assessment as a demonstration that the model within its domain of applicability possesses a satisfactory range of accuracy consistent with the intended application of the model. Rykiel [36] would refer to this type of demonstration as model validation, but we prefer to avoid use of that term (see Grant and Swannack [37]). The domain of applicability of the present model includes environmental conditions generally similar to the Ndumo area of uMkhanyakude district, KwaZulu-Natal Province, South Africa. With reference to similar environmental temperatures, we refer to a range of temperatures within approximately plus or minus four degrees centigrade of those in the Ndumo area. The satisfactory range of accuracy consistent with the intended application of the model we defined as the ability to represent fluctuations in snail population dynamics that exhibit an appropriate temperature-dependent seasonality and that respond in an ecologically reasonable manner to temperature regimes that are warmer than the current temperature regime in the Ndumo area.

### Model application

To investigate how altered water temperatures might affect the production of cercariae and, hence, the transmission of schistosomiasis, we simulated snail population dynamics and the associated production of cercariae using each of five temperature time series. The first temperature time series consisted of temperatures recorded by Manyangadze et al. [3] in the Ndumo area of uMkhanyakude district, KwaZulu-Natal Province, South Africa. The second and third temperature time series consisted of temperatures that had been increased by 1 and 2°C, respectively, which is within the projected range of future temperature rises (1.0–3.5 °C) by 2100 [38]. The fourth and fifth temperature time series consisted of temperatures that had been decreased by 1 and 2 °C, respectively.

### Sensitivity analysis

To assess how sensitive model estimates of cercariae production were to changes in important model parameters, we ran five additional sets of simulations using each of three-time series of temperatures in which we varied each of the five parameters in Table 1 by +10% and -10% of their baseline values.

## Results

### Model evaluation

**Fig 2:**
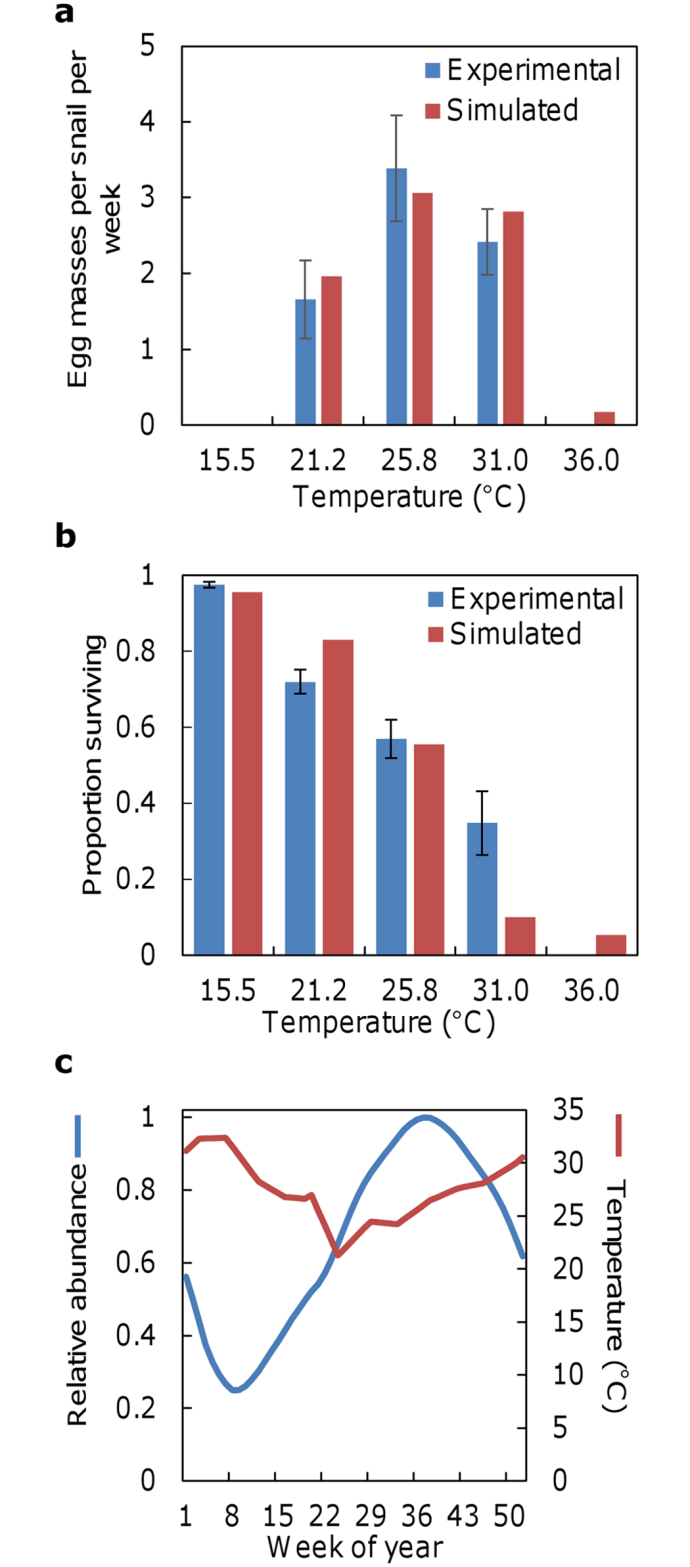
Simulated and experimentally observed [10] snail (a) fecundity (egg masses produced per snail per week over an 11-week period, mean and 95% CI are presented for experimentally observed fecundity) and (b) survival (proportion surviving after 11 weeks, mean and 95% CI are presented for experimentally observed survival) under the indicated temperatures, as well as (c) snail population dynamics (relative abundance) simulated under seasonally varying temperatures representative of generally favorable environmental conditions [3].

Model verification simulations confirmed that the model code generated snail fecundity and survival rates similar to those observed over the range of water temperatures used in the laboratory experiments [10]. For both simulated and experimental output, fecundity was highest at 25.8 °C and decreased at both higher and lower temperatures. No egg masses were produced in the experiments at 15.5 or 36 °C, whereas there was minimal egg production during the simulation at 36 °C (Fig 2a). Model assessment simulations produced reasonable snail population dynamics under seasonally varying temperatures representative of generally favorable environmental conditions [3] (Fig 2c). Simulated populations could not sustain themselves under conditions identified as unfavorably warm (see appendix 2). Both simulated and experimentally observed survival was highest at 15 °C and decreased monotonically in a curvilinear fashion to a low at 36 °C (Fig 2b). Simulated populations fluctuated seasonally in a generally sinusoidal manner, reaching lowest and highest relative abundances in mid-February and mid-September, respectively.

### Model application

**Fig 3:**
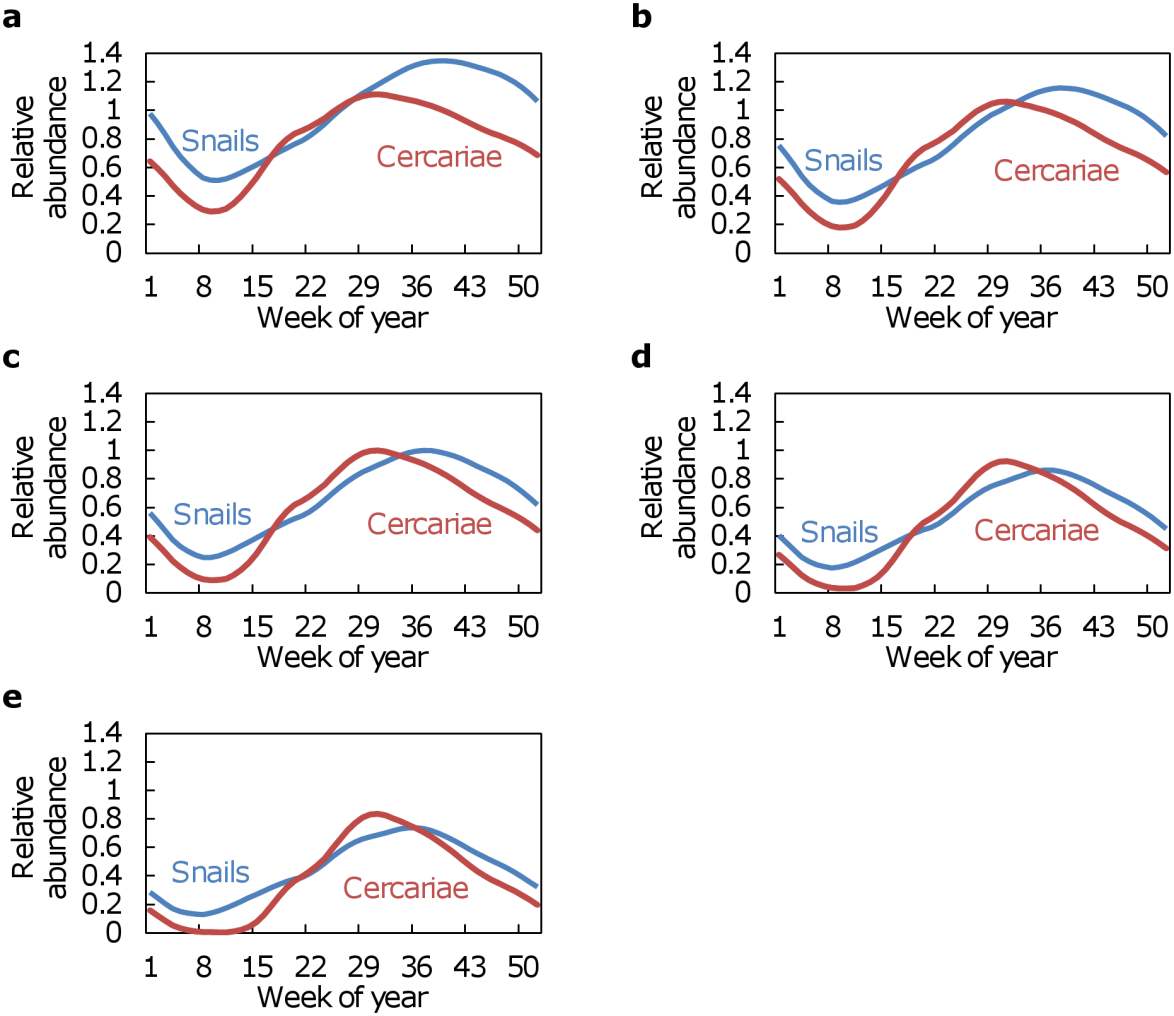
Simulated relative abundances of snails and the associated cercariae released into the environment assuming each of the environmental temperatures in the time series recorded by Manyangadze et al. [3] in the Ndumo area of uMkhanyakude district, KwaZulu-Natal Province, South Africa was (a) decreased by 2 C, (b) decreased by 1 °C, (c) unchanged (d) increased by 1°C, and (e) increased by 2 °C.

Both simulated snail populations and the associated production of cercariae exhibited similar temperature-dependent seasonal fluctuations under each of the first three temperature regimes, with relative abundances of both snails and cercariae decreasing with increasing temperatures (Fig 3). Under all three of these temperature regimes, maximum production of cercariae occurred during week 31, although maximum abundances of snails occurred during week 36. Under simulations in which temperatures were increased by 1 °C and 2 °C, maximum snail abundances were decreased relative to baseline by 14% and 27%, respectively, whereas maximum cercariae productions were decreased by 8% and 17%, respectively. Under the last two temperature regimes, in which temperatures were decreased by 1 °C and 2 °C, maximum snail abundances were increased relative to baseline by 14% and 27%, respectively, whereas maximum cercariae productions were increased by 8% and 17%, respectively.

### Sensitivity analysis

**Fig 4:**
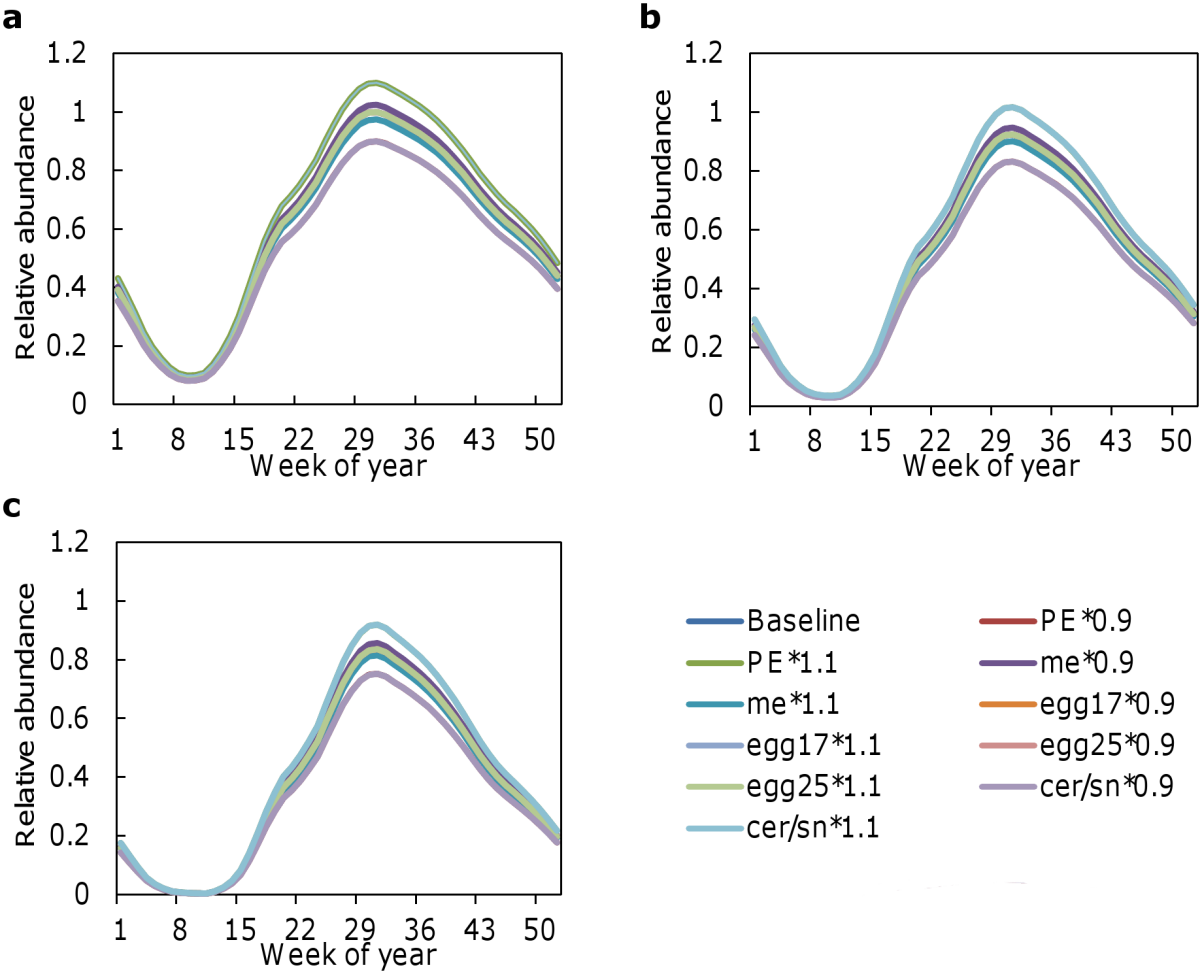
Model estimates of cercariae production during simulations in which each of the five parameters in Table 1 were varied by +10% and -10% of their baseline values assuming (a) seasonally varying temperatures representative of current environmental conditions [3] and assuming temperatures increases of (b) 1 °C and (c) 2 °C.

Model estimates of cercariae production were robust to changes of +10% and -10% relative to baseline in the values of important model parameters (Fig 4). The pattern of seasonal fluctuations in production was not affected by any of the parameter changes nor was the trend of decreasing production with increasing temperatures. The highest peak in cercariae production (≈110% of that occurring during the baseline simulation at current environmental temperatures) occurred under baseline temperatures when ω (number of cercariae released per snail) was increased by 10% (Fig 4a). The lowest peak in cercariae production (≈75% of that occurring during the baseline simulation at current environmental temperatures) occurred under temperatures 2 °C warmer than baseline when ω was decreased by 10% (Fig 4c).

## Discussion

Simulation models that focus on the impact of temperature shifts on the ecology and biology of *Bulinus globosus* and its schistosome parasite, *Schistosoma haematobium* provide key insights into the net effect of climate change on schistosomiasis transmission [25]. Despite growing interest in understanding the linkages between climate change and vector borne diseases, predicting the impact of climate change on diseases such as schistosomiasis remains a challenge [39]. Here, a compartmental simulation model was used to assess the impact of warming on the population dynamics of *B*. *globosus* and the production of *S*. *haematobium*. The current model shows that cercariae production and thus disease risks may be dependent on the density of IH snails. The study also indicates that the abundance of *B*. *globosus* may be affected by changes in the seasonal fluctuation of temperature, an observation that was also made by Woolhouse and Chandiwana [40].

Change in temperature influence snail-trematode systems [30]; it alters snail-parasite interactions [19], affects the biology, ecology and geographical spread of IH snails [4, 7, 17], habitat suitability [3, 27, 41] and intramolluscan parasite development and output [4, 8, 9, 17]. In the current study, temperature had a positive influence on egg mass production. The observed changes in the population of snails due to temperature increase or decrease agree with expectations based on laboratory experiments [8, 19] and field studies [3]. The study indicates that rise in temperature especially in hot areas or during hot seasons may lead to a reduction in the risks of schistosomiasis infection and schistosome transmission may be restricted to isolated areas and seasons which may provide suitable climate for the IH snails [3, 4, 17, 27]. This is further corroborated by the findings of Mangal et al. [24], Morley and Lewis [28] and Ngarakana-Gwasira et al. [11]. While *Schistosoma* infections and the transmission of schistosomiasis may increase in some areas, other areas may have reduced disease incidences due to increased snail mortality [30] and reduced parasite production [42].

Climate change has been predicted to increase temperature by a few degrees [43] and this may impact various ecological processes [29]. However, these changes may vary from place to place [44]. Snails have been observed to acclimatize to a range of temperatures within their thermal limit [23] although mortality increases with temperature [11, 13]. Our simulation model showed a reduction in the amount of cercariae produced at high temperature levels. Furthermore, the simulated stepwise increase in temperature by 1 °C and 2 °C led to an overall reduction in the amount of cercariae produced. The reduction in the amount of cercariae with rise in temperature may be due to temperature induced mortality among snails [8, 21, 45]. Furthermore, physiological processes associated with cercariogenesis have been observed to deteriorate at elevated temperature [28]. This outcome leads to a possible reduction in the risk of schistosomiasis transmission. On the other hand, our study also observed that a reduction in temperature by 1 °C and 2 °C led to an increase in the output of cercariae. According to Manyangadze et al. [3], the habitat suitability for *B*. *globosus* was wider during the cold/dry season when temperatures are low compared to the post-rainy and hot/dry seasons. An increase in cercariae production at transmission sites may increase the risks of *Schistosoma* transmission. These results further indicate that change in temperature may lead to a change in the spatial distribution of schistosomiasis.

The present study has shown the potential impact of temperature-induced changes in the population dynamics and cercariae output of *B*. *globosus* on the transmission of schistosomiasis. Although changes in the life cycle of schistosomiasis and the ecology of *B*. *globosus* have been suggested to depend on temperature [3, 8, 11, 12, 46], water availability and geomorphology will be key in determining the suitability of habitats and transmission of the parasite [3, 47]. Furthermore, change in climate has been predicted to increase the duration, frequency and intensity of droughts [48]. These factors may also affect the survival and reproduction of IH snails as well as lead to their aestivation [49]. Nevertheless, resumption of suitable conditions has been observed to upsurge the population of *B*. *globosus* thus increasing risks of disease transmission [3, 50].

The spatial distribution of IH of schistosomes and their development depends heavily on environmental factors. The current model was developed using data from Ndumo, an area characterized by hot-wet summers, cold-dry winters [51, 52] and high schistosomiasis prevalence [51]. It was also parameterized based on *Bulinus globosus*, the main intermediate host of urogenital schistosomiasis in Southern Africa. Nevertheless, this model can also be applied to other like areas where *B*. *globosus* remains the main intermediate host of *S*. *haematobium*. The model can also be adapted to other snail IHs like *Biomphalaria pfeifferi* and *S*. *mansoni* with the only difference in the temperature ranges and prepatent period.

The results from our simulation showed that the snail population size increased as temperature exceeded 25 °C. This corroborates the findings of Marti [53], Harrison and Shiff [50] and Manyangadze et al. [3] who observed an increase in the population size of *B*. *globosus* as temperature slightly exceeded 25 °C. Our results are also in consonant with the findings of Ngarakana-Gwasira et al. [11] who parameterized a model based on *Bulinus abyssinicus* to determine the potential impact of climate effects on schistosomiasis dynamics in Zimbabwe. Our study further indicates that the temperature range within which fecundity increased coincide with the temperature range (18–28 °C) found to be ideal for transmission of schistosomiasis [11]. This implies that within these temperature ranges and those used in this study in which snail survival was high, warmer temperatures may potentially lead to an increase in the transmission of urogenital schistosomiasis.

Our model representing *B*. *globosus* snails and *S*. *haematobium* cercariae production provides insights into understanding the potential impact of climate change on the population dynamics of the snail and the parasite. The simulation results confirm that both low and high temperature thresholds have important implications for schistosomiasis transmission. While disease risk is likely to be higher in areas with temperatures tolerable to snails, a rise or decrease in temperature outside the tolerable range may alter the spatial distribution of schistosomiasis. The impact of environmental temperature on *B*. *globosus* may not lead to a simple relationship between climate change and the prevalence of urogenital schistosomiasis. Thus, the integration of ecological simulation models based environmental factors within appropriate socio-economic contexts will be a key element in development of effective schistosomiasis control programmes.

## Supporting information

S1 File (DOCX)

S2 File (DOCX)
