# Supplementary material for: Simulation of population dynamics of Bulinus globosus: Effects of environmental temperature on production of Schistosoma haematobium cercariae
Source: PLoS Negl Trop Dis. 2018 Aug 2;12(8):e0006651. doi: 10.1371/journal.pntd.0006651 (PMC6071958; doi:10.1371/journal.pntd.0006651)
Supplement: S1 File — (DOCX) [file pntd.0006651.s001.docx]

Supplementary file 1

Data on snail fecundity and growth was obtained from in-door experiments done on F1 laboratory bred *B. globosus* snails. This data was obtained from the paper done by Kalinda et al. [1]. The snails were kept in an experimental room with a photoperiod of 12:12 h light–dark cycle and fed *ad libitum* on blanched lettuce and supplemented with Tetramin tropical fish food. In total, 405 F1 *B. globosus* snails were used and these were allocated to five different water temperatures. The snails were housed in 2 L containers filled with filtered pond water, each container having 9 snails. The containers housing snails were randomly allocated to fifteen 20 L aquaria in a water bath maintained at predetermined experimental temperatures. The pre-determined water temperatures were: 15.0, 20.0, 25.0, 30.0 and 35.0 °C while the achieved water temperatures (mean ± SE) were: 15.5 ± 0.39, 21.2 ± 0.83, 25.8 ± 0.66, 31.0 ± 0.44 and 36.0 ± 0.35 °C.

The number of egg masses laid were collected and counted over a nine-week period. Egg masses were collected and counted every two days. These were then aggregated to compose a week’s collection. Data on snail mortality across the different experimental temperatures was collected on a daily basis. Snails were only marked as dead if they did not respond to a mechanical stimulus.

Environmental data was adopted from the paper by Manyangadze et al. [2]. In this study, data relating to NDVI and temperature amplitude was calculated from Moderate Resolution Imaging Spectroradiometer (MODIS) reflectance and MODIS minimum and maximum temperature, respectively. This data can be accessed through the International Research Institute for Climate and Society (IRI) data library portal (<http://iridl.ldeo.columbia.edu/SOURCES/>).

1. Kalinda C, Chimbari M, Mukaratirwa S. Effect of temperature on the *Bulinus globosus – Schistosoma haematobium* system. Infectious Diseases of Poverty. 2017; 6: 57.

2. Manyangadze T, Chimbari MJ, Gebreslasie M, Pietro C, Mukaratirwa S. Modelling the spatial and seasonal distribution of suitable habitats of schistosomiasis intermediate host snails using MAXENT in Ndumo area, KwaZulu-Natal Province, South Africa. Parasites & Vectors. 2016; 9 (1): 572.
