# Supplementary material for: Simulation of population dynamics of Bulinus globosus: Effects of environmental temperature on production of Schistosoma haematobium cercariae
Source: PLoS Negl Trop Dis. 2018 Aug 2;12(8):e0006651. doi: 10.1371/journal.pntd.0006651 (PMC6071958; doi:10.1371/journal.pntd.0006651)

Appendix: Simulated relative abundances of snails assuming each of the environmental temperatures in the time series recorded by Manyangadze et al. [3] in the Ndumo area of uMkhanyakude district, KwaZulu-Natal Province, South Africa was altered by the indicated number of degrees centigrade.


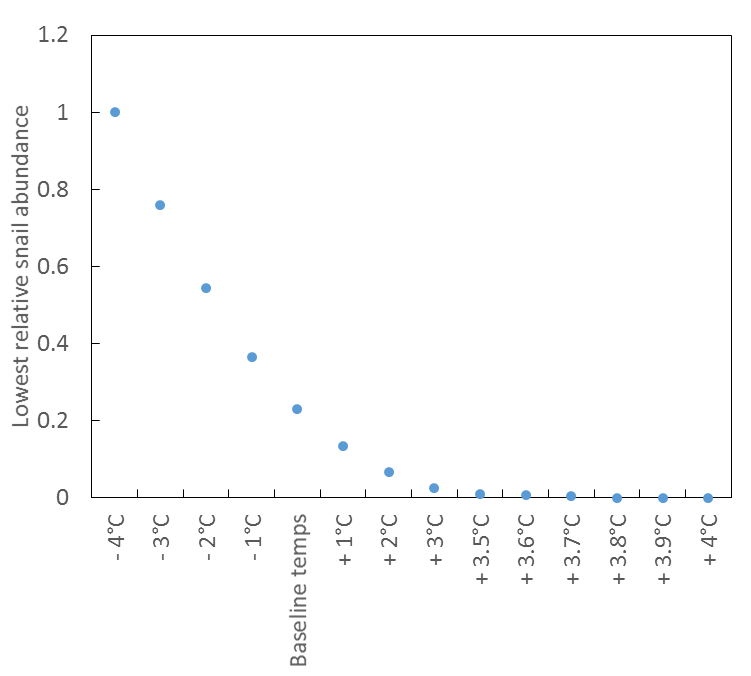

Supplement: S2 File — (DOCX) [file pntd.0006651.s002.docx]
